# Insecticidal and Antifeedant Activities of Plant Essential Oils Against *Spodoptera frugiperda* Larvae

**DOI:** 10.3390/plants15111687

**Published:** 2026-05-29

**Authors:** Taoqi Wu, Xiaolei Xu, Xingzhou Liu, Jianyu Deng, Wenze He, Xunyue Liu, Qiong Rao

**Affiliations:** 1 Zhejiang Key Laboratory of Biology and Ecological Regulation of Crop Pathogens and Insects, College of Advanced Agricultural Sciences, Zhejiang A & F University, Hangzhou 311300, China; wutaoqi@163.com (T.W.); 19012769237@163.com (X.X.); jydeng70@aliyun.com (J.D.); hewenze@zafu.edu.cn (W.H.); 2 Suzhou Academy of Agricultural Sciences, Suzhou 234000, China; lxz8610@163.com

**Keywords:** *Spodoptera frugiperda*, plant essential oil, GC-MS, antifeedant activity, contact toxicity

## Abstract

The fall armyworm (*Spodoptera frugiperda*), a highly destructive invasive Lepidopteran pest, poses a serious threat to global agriculture, particularly maize production. Plant essential oils (EOs) represent a promising class of botanical pesticides owing to their diverse bioactivities and low environmental persistence. In this study, we evaluated the insecticidal and antifeedant activities of 40 commercially available EOs against third-instar *S. frugiperda* larvae. After an initial screening at 4 μL/mL, 10 EOs that caused ≥70% mortality at 72 h were selected for bioassays to estimate LC_50_ value and chemical analysis by GC-MS. Contact toxicity assays showed that geranium EO had the highest activity (LC_50_ = 2.105 μL/mL at 72 h), followed by cypress (2.123 μL/mL) and niaouli (2.391 μL/mL), whereas tea tree EO exhibited the lowest activity (3.592 μL/mL). Antifeedant tests revealed that clove EO caused the strongest feeding deterrence at both 24 h (antifeedant indices AFI = 72.8%) and 48 h (AFI = 63.4%), while most other EOs lost their deterrent effect within 48 h. GC-MS analysis of the 10 active EOs identified a complex mixture of monoterpenes, sesquiterpenes, and oxygenated derivatives; major constituents included D-limonene, 4-terpineol, carvacrol, caryophyllene, and longifolene. These results provide laboratory evidence that several plant EOs, particularly geranium, cypress, niaouli, and clove, possess strong insecticidal and antifeedant activities against *S. frugiperda* larvae, supporting their potential as eco-friendly botanical insecticides.

## 1. Introduction

The fall armyworm (*Spodoptera frugiperda*), belonging to the order Lepidoptera and family Noctuidae, is one of the most destructive invasive pests globally, causing severe damage to agricultural crops worldwide, particularly maize [1,2]. It is a polyphagous insect that infests over 80 host plant species, with a strong preference for Poaceae, although it also damages plants from Brassicaceae, Fabaceae, Asteraceae, and other families [3,4], which can lead to significant yield losses and poses a threat to maize production [5].

However, the extensive use of chemical pesticides has led to the development of widespread resistance to various insecticides in *S. frugiperda*. Recent studies have documented its resistance to multiple classes of chemical insecticides [6]. Plant essential oils (EOs) have emerged as promising botanical pesticide candidates due to their environmental safety, low toxicity, and eco-friendly properties. Research on EOs as botanical pesticides has attracted considerable attention [7]. EOs are complex mixtures of volatile plant compounds [8], primarily consisting of terpenoids, phenols, and alcohols. As secondary metabolites of plants, EOs readily degrade in the environment and do not persist in the air for extended periods, making them safer compared to traditional insecticides [9]. Moreover, their complex and diverse modes of action against pests contribute to a low risk of resistance development, positioning them as a promising novel class of biorational insecticides [10].

It is reported that a variety of biological active constituents effective against insects have been identified in plant extracts and secondary metabolites, including alkaloids, flavonoids, terpenoids, and plant EOs. For example, Liu et al. (2023) tested 11 botanical insecticides against second-instar *S. frugiperda* larvae and found that all exhibited certain insecticidal activities, with eugenol showing the highest toxicity (LD_50_ = 0.191 μg per larva at 72 h) [11]. Liang et al. (2020) reported that limonene (22.05%), which is the main component of *Elsholtzia ciliata* EO, had insecticidal activity against *Tribolium castaneum* [12]. Other studies have showed that lemon EO repels the cabbage looper (*Trichoplusia ni*) [13]. Additionally, origanum EO exhibited a good repellent effect on the mealworm (*Tenebrio molitor*) [14], and basil EO inhibited the oviposition of the tomato leafminer (*Tuta absoluta*) [15]. These findings indicate that EOs and their constituents possess diverse bioactivities, including insecticidal, antifeedant, and repellent effects.

Direct comparison of multiple EOs against *S. frugiperda* under consistent experimental conditions is relatively limited. Furthermore, while larval feeding is the primary cause of crop damage, it has received much less attention than larval mortality. In this study, we evaluated the insecticidal and antifeedant activities of a broad range of EOs against third-instar *S. frugiperda* larvae under laboratory conditions. After an initial screening, the most active EOs were selected for bioassays to determine their LC_50_ values, and their antifeedant activity was quantified by measuring the consumed leaf area in leaf-disk assays. Additionally, the chemical compositions of these active EOs were analyzed by GC-MS to identify their major constituents.

## 2. Results

### 2.1. Toxicity Screening of 40 EOs

**Figure 1:**
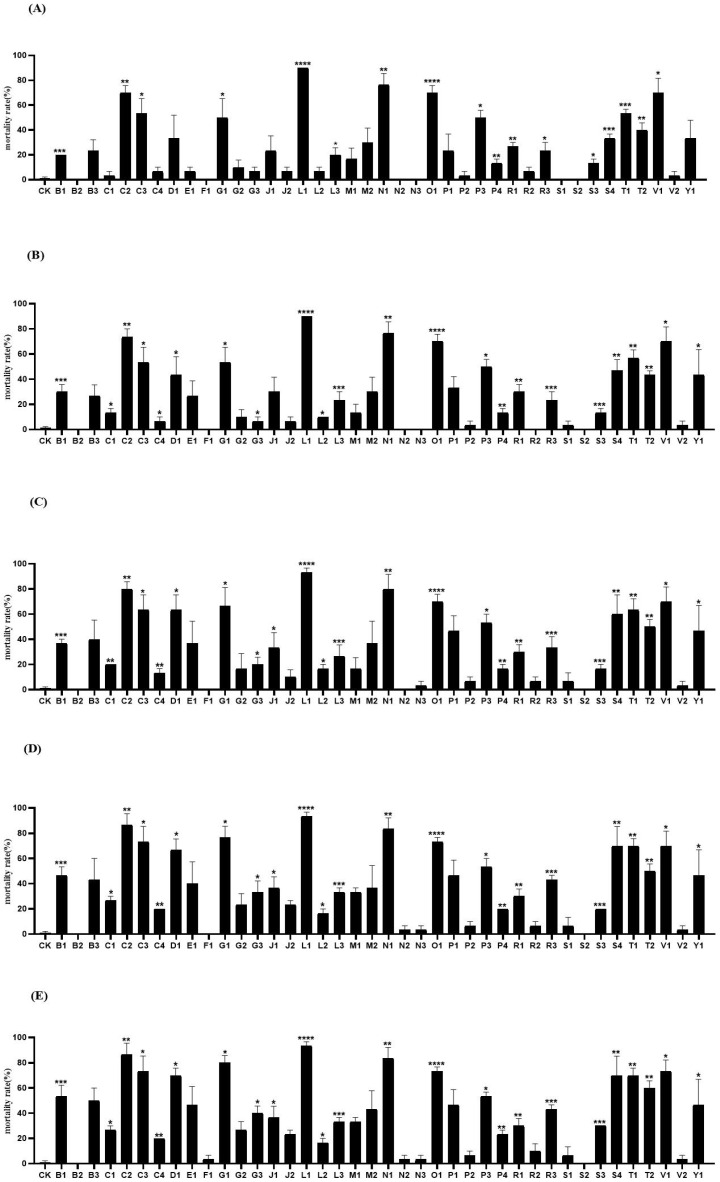
The mortality rate of *Spodoptera frugiperda* larvae after treatment with 40 plant essential oils at 4 μL/mL. Note: (**A**) 6 h, (**B**) 12 h, (**C**) 24 h, (**D**) 48 h, (**E**) 72 h. Data are presented as mean ± SE. * *p* < 0.05; ** *p* < 0.01; *** *p* < 0.001; **** *p* < 0.0001. The EO numbers: B1 (black pepper), B2 (bergamot), B3 (basil), C1 (cinnamon), C2 (clove), C3 (cypress), C4 (chamomile), C5 (citrus), D1 (dill weed), E1 (eucalyptus), F1 (frankincense), G1 (geranium), G2 (ginger), G3 (grapefruit), J1 (juniper berry), J2 (jasmine), L1 (lavender), L2 (lemongrass), L3 (lemon), M1 (myrrh), M2 (melissa), N1 (niaouli), N2 (neroli), N3 (nutmeg), O1 (origanum), P1 (peppermint), P2 (pine needles), P3 (patchouli), P4 (palmarosa), R1 (ravansara), R2 (rose), R3 (rosemary), S1 (sage), S2 (sandalwood), S3 (sweet fennel), S4 (sweet orange), T1 (tea tree), T2 (thyme), V1 (vanilla), V2 (vetiver), Y1 (ylang ylang).

At a concentration of 4 μL/mL, the 40 EOs showed marked differences in contact toxicity against third-instar *S. frugiperda* larvae (Figure 1A–E). At the early exposure time (6 h), lavender (L1) and origanum (O1) EOs caused the highest larval mortality (Figure 1A, *p* < 0.0001). As exposure time increased, more EOs showed potent toxicity. By 72 h, 30 EOs caused significantly higher mortality than the control (*p* < 0.05), and 10 EOs produced mortality rates of 70% or higher. Therefore, these 10 EOs were selected for subsequent concentration–response bioassays. The most effective oils at 72 h, in descending order, were lavender (L1, 93.3 ± 1.7%), clove (C2, 86.6 ± 2.2%), niaouli (N1, 83.3 ± 2.4%), geranium (G1, 80.0 ± 2.1%), origanum (O1, 73.4 ± 3.1%), cypress (C3, 73.3 ± 3.3%), vanilla (V1, 73.0 ± 3.0%), dill (D1, 70.0 ± 2.6%), sweet orange (S4, 70.0 ± 2.8%), and tea tree (T1, 70.0 ± 2.9%).

### 2.2. Toxicity Comparison of Ten Selected EOs

According to the preliminary toxicity screening, 10 EOs with larval mortality ≥ 70% at 72 h were selected for subsequent experiments. Significant differences in toxicity among these EOs were observed, as indicated by their LC_50_ values and corresponding regression equations (Table 1). All ten EOs exhibited varying degrees of contact toxicity, and their insecticidal activity increased over time, as reflected by the decreasing LC_50_ values from 24 h to 72 h. At 24 h, niaouli (N1) and geranium (G1) EOs had the lowest LC_50_ values (both <3 μL/mL). At 48 h, geranium (G1) remained the most toxic, followed by niaouli (N1) and cypress (C3), all with LC_50_ values below 3 μL/mL. In contrast, clove (C2) EO exhibited relatively lower toxicity, with an LC_50_ value of 5.278 μL/mL. By 72 h, five EOs, including geranium (G1), cypress (C3), niaouli (N1), clove (C2), and dill (D1), showed LC_50_ values below 3 μL/mL, indicating strong and stable insecticidal activity. Overall, geranium EO exhibited the strongest and most consistent contact toxicity across all time points.

### 2.3. Antifeedant Activity of Ten Selected EOs

**Figure 2:**
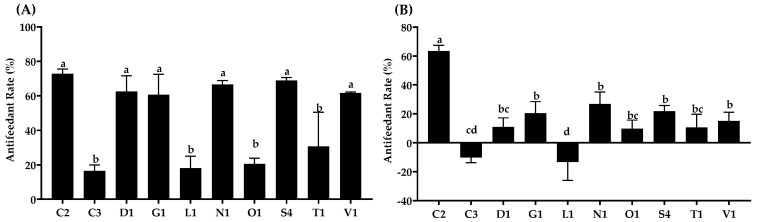
Antifeedant activity of essential oils against *Spodoptera frugiperda* larvae. Note: Data are presented as mean ± SE. Different lowercase letters indicate significant differences among treatments at the same time point (one-way ANOVA followed by Duncan’s multiple range test, *p* < 0.05). Note: (**A**) After 24 h; (**B**) After 48 h of EOs treatments. The EO numbers: clove (C2), cypress (C3), dill (D1), geranium (G1), lavender (L1), niaouli (N1), origanum (O1), sweet orange (S4), tea tree (T1), vanilla (V1).

At 24 h, the control group consumed 19.03 ± 2.11 mm^2^ of leaf area. Among all EOs, clove C2 EO showed the highest antifeedant effect, with a consumed area of 5.17 ± 0.50 mm^2^ and an antifeedant indices (AFI) of 72.83 ± 2.64%, followed by sweet orange S4 EO (5.93 ± 0.33 mm^2^, AFI 68.87 ± 1.71%), niaouli N1 EO (6.37 ± 0.45 mm^2^, AFI 66.53 ± 2.38%), dill D1 EO (7.13 ± 1.74 mm^2^, AFI 62.54 ± 9.12%), vanilla V1 EO (7.30 ± 0.11 mm^2^, AFI 61.64 ± 0.58%), and geranium G1 EO (7.50 ± 2.27 mm^2^, AFI 60.62 ± 11.94%). All these six EOs had AFI values exceeding 60% (Figure 2), indicating strong feeding deterrence. The remaining EOs displayed substantially lower antifeedant activity, with AFI values below 31%. In descending order, tea tree T1 EO consumed 13.20 ± 3.76 mm^2^ (AFI 30.65 ± 19.76%), origanum O1 EO consumed 15.13 ± 0.66 mm^2^ (AFI 20.48 ± 3.47%), lavender L1 EO consumed 15.60 ± 1.33 mm^2^ (AFI 18.04 ± 7.01%), and cypress C3 EO consumed 15.90 ± 0.67 mm^2^ (AFI 16.47 ± 3.54%).

At 48 h, the control group’s consumed leaf area increased to 53.23 ± 1.60 mm^2^. The antifeedant index of all EOs was lower than at 24 h. Clove C2 EO retained the highest AFI, with a consumed area of 19.49 ± 2.10 mm^2^ and an AFI of 63.38 ± 3.94%, which was significantly higher than all other EOs (*p* < 0.05, one-way ANOVA followed by Duncan’s test). The next most effective EOs in descending order of AFI were niaouli N1 EO (39.03 ± 4.51 mm^2^, AFI 26.68 ± 8.46%), sweet orange S4 EO (41.68 ± 2.16 mm^2^, AFI 21.71 ± 4.07%), geranium G1 EO (42.37 ± 4.27 mm^2^, AFI 20.40 ± 8.02%), vanilla V1 EO (45.23 ± 3.15 mm^2^, AFI 15.02 ± 5.93%), tea tree T1 EO (47.56 ± 4.80 mm^2^, AFI 10.66 ± 9.01%), dill D1 EO (47.43 ± 3.39 mm^2^, AFI 10.90 ± 6.38%), and origanum O1 EO (48.09 ± 3.19 mm^2^, AFI 9.67 ± 5.98%). However, none of these EOs had consumed areas statistically different from the control. The remaining EOs, cypress C3 EO (58.70 ± 1.88 mm^2^) and lavender L1 EO (60.31 ± 6.83 mm^2^), exhibited negative AFI values, suggesting a possible reversal from weak deterrence to slight feeding stimulation. These results demonstrate that only clove C2 EO provided both immediate and persistent antifeedant activity, whereas the other effective EOs acted rapidly but lost most of their efficacy within 48 h.

### 2.4. Selective Antifeedant Activity of EOs Against S. frugiperda Larvae

**Figure 3:**
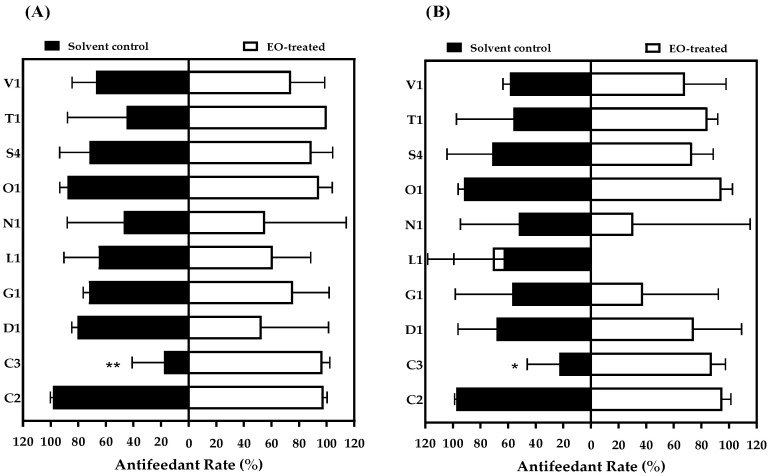
Antifeedant activity of essential oils against *Spodoptera frugiperda* larvae. Note: The data in the figure are all presented as mean ± SE. * *p* < 0.05; ** *p* < 0.01. (**A**) After 24 h; (**B**) After 48 h. The EO numbers: clove (C2), cypress (C3), dill (D1), geranium (G1), lavender (L1), niaouli (N1), origanum (O1), sweet orange (S4), tea tree (T1), vanilla (V1).

The selective antifeedant activity of the ten EOs was evaluated using a selective leaf-disc assay, and the selective antifeedant index (SAFI) on EO-treated versus control discs at 24 h and 48 h is shown in Figure 3. At 24 h, tea tree (T1) EO exhibited the strongest antifeedant activity, with a SAFI of 100% on treated leaf-discs, while the corresponding control leaf-discs had a SAFI of only 44.97 ± 20.16% (*p* < 0.01). At 48 h, T1 maintained a high SAFI of 84.43 ± 3.50% on treated discs, compared to a control SAFI of 56.17 ± 19.40%. Cypress (C3) EO also demonstrated high activity at 24 h, with a treated SAFI of 96.93 ± 2.51% versus a control SAFI of 17.82 ± 10.88%. At 48 h, C3 remained effective, with a treated SAFI of 87.49 ± 4.66% and a control SAFI of 22.8 ± 10.98% (the lowest at 48 h).

Several EOs exhibited a fumigant effect, where both treated and control discs showed high SAFI values due to volatile cross-contamination. At 24 h, clove (C2) and origanum (O1) EOs showed high treated SAFI values (C2: 97.75 ± 1.18%; O1: 94.56 ± 4.44%), and their respective control discs also had high SAFI values (C2 control: 98.29 ± 0.89%; O1 control: 87.82 ± 2.62%), indicating that volatiles from the treated disc suppressed feeding on the adjacent untreated disc. Similarly, sweet orange (S4) EO achieved a treated SAFI of 89.11 ± 7.14% at 24 h, while its control discs showed a high SAFI of 71.85 ± 10.2%. Geranium (G1) EO showed moderate activity at 24 h (treated 75.63 ± 12.32%, control 72.16 ± 1.95%), but this effect was transient. At 48 h, the fumigant effect persisted for C2 (treated 95.14 ± 2.91%, control 97.56 ± 0.55%), O1 (94.56 ± 3.71%, control 91.99 ± 1.98%), and partially for S4 (treated 73.19 ± 7.18%, control 71.44 ± 15.46%), whereas G1 lost its activity (treated 37.74 ± 25.69%, control 57.01 ± 19.4%).

In contrast, cypress (C3) and tea tree (T1) EOs maintained a true repellent/antifeedant effect. At 48 h, C3 retained a strong SAFI of 87.49 ± 4.66% (control 22.8 ± 10.98%), and T1 remained effective with a SAFI of 84.43 ± 3.50% (control 56.17 ± 19.4%). However, lavender (L1) EO showed a negative SAFI at 48 h, indicating feeding stimulation (control SAFI 63.2 ± 16.99%). Niaouli (N1) and geranium (G1) also lost most of their deterrent activity by 48 h.

### 2.5. GC-MS Analysis of Ten Selected EOs

The chemical compositions of the 10 active EOs were analyzed by GC-MS. The identified constituents and their relative abundances (calculated by GC-MS peak area normalization) are listed in Table 2 (detailed in Supplementary Tables S1–S10). Among them, (−)-Isocaryophyllene was detected in five EOs, with concentrations of 73.02% in clove (C2), 30.50% in geranium (G1), 16.64% in vanilla (V1), 6.00% in cypress (C3), and 1.81% in tea tree (T1). Other components found in five EOs included Linalool, (+)-δ-Cadinene, and α-Caryophyllene. Among these, (+)-δ-cadinene was the predominant component in cypress (C3) at 21.26% and linalool was detected in lavender (L1) at 22.20%. D-Limonene, p-Cymene, α-Terpineol, Eucalyptol occurred in four EOs. D-limonene exceeded 70% in sweet orange (S4) as its dominant constituent, and 24.77% in dill (D1).

Compounds shared by three EOs included (+)-Longifolene, L-(−)-Camphor, α-Phellandrene, β-Pinene, Aromadendrene, (−)-Caryophyllene oxide. (+)-longifolene was the dominant component in vanilla (V1) at 43.83%, and also exceeded 10.00% in cypress (C3) and geranium (G1). γ-terpinene accounted for 13.58% in niaouli (N1) (its second most abundant compound) and surpassed 2.00% in tea tree (T1). The remaining components were unique to specific EOs, including (±)-4-terpineol (the predominant component in niaouli (N1) [48.46%] and tea tree (T1) [28.96%]); triacetin (the main component of lavender (L1) [46.51%]); D-(+)-carvone (the primary constituent of dill (D1) [56.16%]); and carvacrol (the predominant compound in origanum EO (O1) [61.10%]).

## 3. Discussion

The present study systematically evaluated the insecticidal and antifeedant activities of 40 plant EOs against *S. frugiperda* and identified several EOs with strong bioactivity. The observed variability in toxicity among different EOs is consistent with previous findings that the efficacy of plant-derived products largely depends on their chemical composition and plant origin [9,36,37].

Toxicity bioassays showed that several EOs exhibited strong insecticidal activity, with LC_50_ values decreasing over time. This time-dependent increase in toxicity has been widely reported and is generally attributed to cumulative exposure effects and enhanced penetration of volatile compounds into insect tissues [36,38,39]. Similar trends have also been observed for EOs tested against other lepidopteran pests [10,40]. Among the 40 EOs screened, 10 caused more than 70% mortality at 72 h. Geranium EO showed the strongest contact toxicity (LC_50_ = 2.105 μL/mL), followed by cypress (2.123 μL/mL) and niaouli (2.391 μL/mL). The high toxicity of geranium EO may be attributed to its high contents of (−)-isocaryophyllene (30.50%), (+)-longifolene (15.87%) and isosativene (8.59%), as volatile sesquiterpenes and oxygenated compounds are known to contribute significantly to insecticidal activity [41,42]. Niaouli EO contained a high proportion of (±)-4-terpineol (48.46%) and γ-terpinene (13.58%), which have been reported to possess strong contact and fumigant toxicity against agricultural pests [43,44]. Most EOs showed lower toxicity at 24 h but significantly increased mortality after 48–72 h, indicating that EOs act relatively slowly compared with synthetic insecticides. This pattern is common in botanical pesticides and reflects a mode of action involving metabolic disruption rather than rapid neurotoxic paralysis [9,10]. Clove EO exhibited moderate toxicity at 24–48 h but achieved an LC_50_ of 2.534 μL/mL at 72 h, likely due to eugenol (12.43%), which is highly toxic to *S. frugiperda* larvae [11]. These results collectively indicate that insecticidal activity of EOs depends on both exposure time and chemical composition.

In addition to causing mortality, many EOs significantly inhibited larval feeding, as reflected by AFI and reduced leaf consumption. Feeding deterrence is a key mechanism in botanical pest control, as it directly reduces plant damage even when lethal effects are moderate [9,13]. Previous studies have shown that EOs can disrupt insect feeding behavior by affecting gustatory receptors and altering sensory perception [36,40]. In non-selective tests, clove EO showed the strongest antifeedant effect (AFI = 72.8%), followed by sweet orange (68.8%), niaouli (66.5%) and vanilla (61.6%). The strong antifeedant activity of clove EO may be related to its high content of (−)-isocaryophyllene (73.02%) and eugenol (12.43%), while tea tree EO contained (±)-4-terpineol (28.96%) and α-terpineol (5.34%), known repellents and antifeedants against lepidopteran larvae [43,45]. Sweet orange EO, rich in D-limonene (78.25%), also significantly inhibited feeding, consistent with reports that monoterpene hydrocarbons can deter insect feeding [12,46].

The selective feeding assay further provided insight into the behavioral mechanisms of the tested EOs. Taken together, among the ten tested EOs, cypress (C3) and tea tree (T1) demonstrated the most reliable and persistent selective antifeedant activity, while C2, O1, and S4 acted primarily via volatile-mediated fumigation, and L1 showed a transient attractant effect at 48 h. The strong repellent/antifeedant effect of C3 and T1, evidenced by high SAFI values on treated discs and low SAFI on adjacent control discs, indicates that larvae can actively detect and avoid these EOs [43,45]. In contrast, the fumigant effect of C2, O1, and S4—where both treated and control discs exhibited high SAFI values (>70%)—suggests that their volatile components saturate the local atmosphere, which may be useful in closed environments such as greenhouses or storage facilities [44]. The negative SAFI observed for L1 at 48 h, indicating feeding stimulation, is unexpected but not unprecedented; similar biphasic (repellent then attractant) effects have been reported for certain plant volatiles at different concentrations or exposure times, possibly due to olfactory adaptation or a shift in the insect’s physiological state.

GC-MS analysis revealed that the major components of the selected EOs were predominantly monoterpenes and, to a lesser extent, sesquiterpenes. Compounds such as D-limonene, linalool, eucalyptol, terpinen-4-ol, and citronellal were widely detected. These compounds have frequently been reported to exhibit insecticidal or repellent activities against various pest species [9,13,36,47]. Oxygenated monoterpenes are considered particularly important due to their higher polarity and biological reactivity [47]. Synergistic interactions among multiple components in EOs may enhance overall bioactivity. Similar synergistic effects have been widely reported, where complex mixtures act on multiple targets and delay resistance development [9,10,12]. Therefore, the insecticidal and antifeedant activities of EOs cannot be fully explained by individual dominant components but arise from the combined effects of multiple constituents. It is important to emphasize that EOs are complex mixtures, and their biological activity cannot be attributed to a single compound. Some compounds were present at high concentrations but restricted to specific EOs, while others were more widely distributed. Furthermore, sesquiterpenes such as caryophyllene [28], cadinene [31], and aromadendrene [30] were also identified at lower concentrations, potentially playing secondary or synergistic roles [37,40].

Despite these encouraging findings, several limitations of this study should be addressed in future research. Regarding chemical analysis, the compositions presented here are based on GC-MS peak area normalization. While this method is commonly used for comparative characterization of essential oils in bioactivity screening, absolute quantification of individual constituents would require GC-FID analysis with appropriate response factors, as recommended by Bicchi et al. (2008) [48]. Furthermore, the detailed mechanisms underlying the observed toxicity and antifeedant activity, such as effects on enzyme systems, neural transmission, and detoxification metabolism, were not explored and warrant further investigation. From a practical perspective, all bioassays were conducted under laboratory conditions, and field efficacy requires further validation, as environmental factors such as temperature, humidity, and UV radiation might accelerate volatilization and degradation, reducing persistence [49,50]. The development of microemulsions, nanoformulations, or encapsulation systems would be beneficial to improve stability, sustained release, and field applicability [41,49]. Finally, safety to natural enemies, pollinators, and other non-target organisms were not evaluated in this study; some EOs could pose risks to beneficial arthropods at effective concentrations [51,52].

## 4. Materials and Methods

### 4.1. Insects

*Spodoptera frugiperda* were originally collected from maize plants in Muqiao Village, Hangzhou, China, and reared in the laboratory for multiple generations without insecticide exposure. The species identity was confirmed according to previously reported morphological and molecular descriptions in our lab [53]. The larvae used in the experiments were obtained from this laboratory-maintained colony. The colony was maintained on an artificial diet at 28 ± 1 °C, 65 ± 5% relative humidity, and a 16:8 h (L:D) photoperiod. The diet consisted of wheat bran (300 g), soybean protein powder (100 g), maize flour (200 g), agar (15 g), yeast powder (30 g), sorbic acid (2.5 g), antiradical acid (10 g), methyl p-hydroxybenzoate (5 g), vitamins, and trace elements, prepared in distilled water and sterilized at 121 °C. The diet was stored at 4 °C. Maize plants (Jiayu 538) were grown in a greenhouse at Zhejiang A&F University.

### 4.2. Plant Essential Oils

The EOs used in this study were commercially purchased and used as received without further extraction or purification from Fengya Pharmaceutical Technology Co., Ltd, Guangzhou, China. The EOs included the following: B1 (black pepper), B2 (bergamot), B3 (basil), C1 (cinnamon), C2 (clove), C3 (cypress), C4 (chamomile), D1 (dill weed), E1 (eucalyptus), F1 (frankincense), G1 (geranium), G2 (ginger), G3 (grapefruit), J1 (juniper berry), J2 (jasmine), L1 (lavender), L2 (lemongrass), L3 (lemon), M1 (myrrh), M2 (melissa), N1 (niaouli), N2 (neroli), N3 (nutmeg), O1 (origanum), P1 (peppermint), P2 (pine needles), P3 (patchouli), P4 (palmarosa), R1 (ravansara), R2 (rose), R3 (rosemary), S1 (sage), S2 (sandalwood), S3 (sweet fennel), S4 (sweet orange), T1 (tea tree), T2 (thyme), V1 (vanilla), V2 (vetiver), and Y1 (ylang).

### 4.3. Screening of EOs and Bioassay on S. frugiperda Larvae

Each of the 40 EOs were diluted with 0.1% Tween-80 solution (Macklin Biochemical Technology Co., Ltd., Shanghai, China) to a concentration of 4 μL/mL. The insecticidal activity was evaluated using a larval immersion method (dipping bioassay) [54]. After being air-dried, they were transferred to Petri dishes containing fresh feed and placed in an insect rearing room. Larval mortality in each treatment was recorded at 6, 12, 24, 48, and 72 h post-treatment. A 0.1% Tween-80 solution was used as the control. Each treatment consisted of ten larvae, with three indespendent replicates per EO.

Based on the preliminary screening results, ten EOs that induced over 72% mortality after 72 h were selected for further bioassay. These EOs were serially diluted with 0.1% Tween-80 solution to five different concentration gradients. Each treatment was tested against healthy third-instar larvae, with three replicates per concentration.

### 4.4. Antifeedant Activity Assay

The antifeedant activity of the EOs against the larvae was evaluated using a leaf-disk method. The assay was performed following the method described by Escoubas et al. (1993) with slight modifications [55]. Fresh maize leaves at the early growth stage were collected and cut into 30 mm diameter discs for subsequent experiments. An EO microemulsion was prepared at a concentration of 4 μL/mL. The leaf discs were immersed in the microemulsion for 10 s, then removed and air-dried at room temperature. Each treated leaf disc was separately placed into a Petri dish containing 1% agar. The third-instar larvae starved for 4 h were selected and placed on each leaf disc, with one larva per dish. For each treatment, three replicates were set up, each replicate consisting of 10 to 20 dishes (i.e., 10 to 20 larvae). At 6 h after exposure, larval survival was checked; dead larvae were removed, and only live larvae were kept. Each replicate was required to have at least five live larvae for subsequent observations. The treatment with a 0.1%Tween-80 solution was used as the control.

After 24 and 48 h of exposure, the consumed leaf area was measured under a stereomicroscope (Nikon SMZ25, Nikon Corporation, Tokyo, Japan)). The antifeedant indices (AFI) were calculated as:

AFI (%) = [(C − T)/C] × 100

where C represents the leaf area consumed in the control group, and T represents the leaf area consumed in the EO-treated group.

### 4.5. Selective Feeding Assay

The selective antifeedant activity was evaluated using a choice leaf-disc method. Fresh maize leaves at the early growth stage were collected and cut into 30 mm diameter leaf-discs. The EOs was diluted with 0.1% Tween-80 solution to a concentration of 4 μL/mL. Leaf-discs were immersed in the EO microemulsion for 10 s, then removed and air-dried at room temperature. Control leaf-discs were treated with 0.1% Tween-80 solution only. Each Petri dish (90 mm diameter) was lined with 1% agar to maintain humidity. Two EO-treated and two control leaf-discs were placed alternately in each Petri dish. Third-instar larvae starved for 4 h were placed individually into each dish (one larva per dish). Each treatment consisted of five dishes, and the entire experiment was replicated three times independently. At 24 h and 48 h after exposure, the consumed leaf area on each disc was measured under a stereomicroscope (LEICA EZ4). The selective antifeedant index (SAFI) was calculated as:

SAFI (%) = [(C − 2T_S_)/C] × 100

where C is the leaf total area consumed on control Peri dish (with 4 control leaf-discs), and T_S_ is the leaf area consumed on EO-treated discs or control-treated discs in the same Peri dish. A SAFI value of 100% indicates complete feeding deterrence, 0% indicates no effect, and a negative value indicates feeding stimulation.

### 4.6. GC-MS Analysis

Each of the 10 selected EOs were diluted in chromatographic-grade n-hexane to a concentration of 1 μL/mL. The solutions were filtered through a 0.22 μm organic membrane filter prior to analysis and transferred into injection vials. GC–MS analysis was performed using an Rtx-5MS capillary column (30 m × 0.25 mm × 0.25 μm). The injection volume was 1 μL with a split ratio of 10:1. High-purity helium was used as the carrier gas at a constant flow rate of 1.0 mL/min. The injection port temperature was set at 250 °C. The oven temperature program was as follows: 50 °C for 3 min, increased at 5 °C/min to 100 °C for 3 min, then to 150 °C for 3 min, then to 200 °C for 3 min, and finally to 230 °C for 10 min. Mass spectrometry was conducted using electron ionization (EI) at 70 eV, with an ion source temperature of 230 °C and an interface temperature of 220 °C. The mass scan range was *m*/*z* 35–650.

The components were identified by comparing mass spectra with the NIST library. Retention indices (RI) were obtained from Adams’ EOs identification manual [56]. Each EO was analyzed in triplicate. The relative percentage of each component was calculated [57] by peak area normalization using the following equation:

$$P_{i}=\frac{A_{i}}{\sum A_{i}}\times 100\%$$

where *P_i_* represents the relative percentage of component *i*, *A_i_* is the peak area of the individual compound, and $\sum A_{i}$ denotes the total peak area of all detected components. Quantification note: The relative percentages presented above were calculated by GC-MS peak area normalization.

### 4.7. Data Analysis

Statistical analyses were performed using SPSS 26.0. LC_50_ values and their 95% confidence limits were calculated by probit analysis based on mortality data from all tested concentrations (including the control). Data are presented as mean ± standard error (SE). One-way ANOVA followed by Duncan’s multiple range test was used.

## 5. Conclusions

Among 40 plant essential oils tested at 4 μL/mL, 10 caused ≥70% mortality of third-instar *S. frugiperda* larvae at 72 h. Geranium EO showed the highest contact toxicity (LC_50_ = 2.105 μL/mL), followed by cypress (2.123 μL/mL) and niaouli (2.391 μL/mL). Clove EO exhibited the strongest antifeedant effect at 24 h (AFI = 72.8%) and was the only oil that retained significant deterrence at 48 h (AFI = 63.4%). GC-MS analysis revealed the major constituents as relative abundances calculated by peak area normalization, including D-limonene (78.25% in sweet orange), 4-terpineol (48.46% in niaouli, 28.96% in tea tree), carvacrol (61.10% in origanum), caryophyllene (73.02% in clove), and longifolene (43.84% in vanilla). These results demonstrate that several commercially available EOs, particularly geranium, cypress, niaouli, and clove, possess potent insecticidal and antifeedant activities against *S. frugiperda* larvae, supporting their potential as biorational pesticides.

## Figures and Tables

**Table 1:** Contact toxicity of ten plant essential oils against *Spodoptera frugiperda* larvae.

Essential Oils	24 h		48 h		72 h	
	LC_50_(μL/mL) 95%CL	Regression Equation	LC_50_(μL/mL) 95%CL	Regression Equation	LC_50_(μL/mL) 95%CL	Regression Equation
clove	6.265	y = −2.06 + 2.72x	5.278	y = −1.07 + 1.47x	2.534	y = −0.43 + 1.09x
(C2)	4.624–10.954		3.818–8.724		1.589–4.559	
cypress	3.423	y = −0.98 + 1.75x	2.797	y = −0.83 + 1.58x	2.123	y = −0.64 + 1.91x
(C3)	1.078–67.794		0.829–19.418		0.393–12.800	
dill	3.544	y = −1.21 + 2.16x	3.234	y = −1.13 + 2.22x	2.885	y = −1.23 + 2.72x
(D1)	2.806–4.445		2.555–4.040		2.335–3.524	
geranium	2.642	y = −1.30 + 3.08x	2.322	y = −0.94 + 2.50x	2.105	y = −0.68 + 2.10x
(G1)	0.970–8.039		0.799–7.270		0.625–7.481	
lavender	4.282	y = −1.41 + 2.23x	3.837	y = −1.36 + 2.34x	3.561	y = −1.34 + 2.41x
(L1)	3.393–5.724		3.070–4.980		2.841–4.626	
niaouli	2.539	y = −0.87 + 2.07x	2.440	y = −0.92 + 2.30x	2.391	y = −0.95 + 2.45x
(N1)	1.915–3.237		1.879–3.063		1.859–2.983	
origanum	5.163	y = −0.97 + 1.39x	3.14	y = −0.80 + 1.65x	2.832	y = −0.77 + 1.73x
(O1)	3.485–9.975		2.286–4.700		2.094–4.051	
sweet orange	3.882	y = −1.01 + 1.64x	3.683	y = −1.01 + 1.77x	3.227	y = −0.93 + 1.69x
(S4)	2.829–5.304		2.665–5.016		2.386–4.250	
tea tree	4.118	y = −1.16 + 1.91x	3.794	y = −1.12 + 1.95x	3.592	y = −1.08 + 1.89x
(T1)	3.080–5.520		2.884–5.028		2.705–4.716	
vanilla	3.725	y = −1.29 + 2.24x	3.226	y = −1.08 + 2.12x	3.155	y = −1.06 + 2.13x
(V1)	2.964–4.662		2.523–4.061		2.465–3.967	

**Table 2:** Main components and relative abundance by GC-MS of ten plant essential oils.

No	Chemical Components	Relative Abundance (%) by GC-MS of Essential Oils										Chemical Class	Lepidoptera Pest	Insecticidal Activity	Ref.
		C2	C3	D1	G1	L1	N1	O1	S4	T1	V1				
1	D-Limonene			24.77		0.22			78.25		0.81	Monoterpene	*Spodoptera frugiperda*	Larvicidal/contact	[16]
2	(±)-4-Terpineol						48.46			28.96		Monoterpene	*Plutella xylostella*	Contact & fumigant	[17]
3	Carvacrol							61.10				Monoterpene	*Helicoverpa armigera*	Larvicidal/ovicidal	[18]
4	D-(+)-Carvone			56.16								Monoterpene	*Galleria mellonella*	Larvicidal/adulticidal	[19]
5	Linalool				1.74	22.20	4.23	4.16	0.45			Monoterpene	*Helicoverpa armigera*	Contact toxicity	[20]
6	p-Cymene			3.46			7.63	12.63		0.42		Monoterpene	*Helicoverpa armigera*	Larvicidal/ovicidal	[18]
7	γ-Terpinene						13.58			2.04		Monoterpene	*Helicoverpa armigera*	Larvicidal/ovicidal	[18]
8	Citronellal										13.45	Monoterpene	*Plodia interpunctella*	Fumigant	[21]
9	α-Terpineol				0.41		3.71			5.34	1.96	Monoterpene	*Spodoptera litura*	Antifeedant	[22]
10	Citronellol				6.56						2.62	Monoterpene	*Spodoptera frugiperda*	Toxicity/repellent	[23]
11	L-(−)-Camphor					1.22	5.72			0.86		Monoterpene	*Tuta absoluta*	Fumigant/repellent	[24]
12	Terpinyl acetate					4.68				1.76		Monoterpene	*Cydia pomonella*	Enhances pheromone trap	[25]
13	Thymol							5.03				Monoterpene	*Spodoptera litura*	Antifeedant	[22]
14	α-Phellandrene			3.19			0.47	0.13				Monoterpene	*Pieris brassicae*	Antifeedant/toxicity	[26]
15	Eucalyptol					1.16	1.08	0.26		1.24		Monoterpene	*Acrobasis advenella*	Fumigant	[27]
16	β-Pinene						1.09	1.01	0.25			Monoterpene	*Acrobasis advenella*	Fumigant	[27]
17	(−)-Isocaryophyllene	73.02	6.00		30.50					1.81	16.64	Sesquiterpene	*Spodoptera litura*	Growth inhibition/mortality	[28]
18	(+)-Longifolene		11.80		15.87						43.84	Sesquiterpene	*Trabala vishnou gigantina*	Olfactory attraction	[29]
19	(−)-Aromadendrene									29.90		Sesquiterpene	*Hyphantria cunea*	Repellent (potentially)	[30]
20	(+)-δ-Cadinene	0.77	21.26		0.58					0.65	1.21	Sesquiterpene	*Spodoptera exigua*	Antifeedant	[31]
21	α-Caryophyllene	7.50	2.13		1.06					1.61	2.92	Sesquiterpene	*Spodoptera litura*	Growth inhibition/mortality	[28]
22	γ-Elemene		9.69					1.51				Sesquiterpene	*Spodoptera litura*	Contact toxicity	[32]
23	Aromadendrene	0.41	6.37				0.59					Sesquiterpene	*Hyphantria cunea*	Repellent (potentially)	[30]
24	Cedrol		4.46									Sesquiterpene	*Chilo suppressalis*	Oviposition attraction	[33]
25	(−)-Caryophyllene oxide	2.86			1.35						0.65	Sesquiterpene	*Spodoptera frugiperda*	Larval attraction	[34]
26	Eugenol	12.43										Non-terpenoid	*Galleria mellonella*	Insecticidal	[35]

Note: The chemical constituents listed in this table were selected from the GC-MS analysis results (see Supplementary Tables S1–S10) based on the following criteria: (i) a relative abundance greater than 3% in the corresponding essential oil, or (ii) occurrence in three or more of the tested essential oils. In addition, only compounds with previously documented insecticidal, repellent, or antifeedant activities against Lepidoptera pest in the scientific literature are included. EO numbers: clove (C2), cypress (C3), dill (D1), geranium (G1), lavender (L1), niaouli (N1), origanum (O1), sweet orange (S4), tea tree (T1), vanilla (V1).

## Data Availability

The data presented in this study are available on request from the corresponding author. The data are not publicly available due to privacy restriction.

## Supplementary Materials

The following supporting information can be downloaded at: https://www.mdpi.com/article/10.3390/plants15111687/s1, Table S1: Chemical constituents and their relative abundance by GC-MS in clove C2 essential oil; Table S2: Chemical constituents and their relative abundance by GC-MS in cypress C3 essential oil; Table S3: Chemical constituents and their relative abundance by GC-MS in dill D1 weed essential oil; Table S4: Chemical constituents and their relative abundance by GC-MS in geranium G1 essential oil; Table S5: Chemical constituents and their relative abundance by GC-MS in lavender L1 essential oil; Table S6: Chemical constituents and their relative abundance by GC-MS in niaouli N1 essential oil; Table S7: Chemical constituents and their relative abundance by GC-MS in origanum O1 essential oil; Table S8: Chemical constituents and their relative abundance by GC-MS in vanilla V1 essential oil; Table S9: Chemical constituents and their relative abundance by GC-MS in tea tree T1 essential oil; Table S10: Chemical constituents and their relative abundance by GC-MS in sweet orange S4 essential oil.
