# Supplementary material for: Insecticidal and Antifeedant Activities of Plant Essential Oils Against Spodoptera frugiperda Larvae
Source: Plants (Basel). 2026 May 29;15(11):1687. doi: 10.3390/plants15111687 (PMC13259549; doi:10.3390/plants15111687)
Supplement: Supplementary file 1 [file plants-15-01687-s001.zip › plants-4218541-supplementary.pdf]

Appendix tables

Table S1. Chemical constituents and their relative abundance by GC-MS in clove C2 essential oil

RT/min	Compound	RI	Relative abundance (%)
22.77	α-Cubebene	1345	0.25
23.06	Phenol, 2-methoxy-3-(2-propenyl)-	-	12.43
23.65	α-Copaene	1374	1.16
25.13	(-)-Isocaryophyllene	1408	73.02
25.30	Aromadendrene	1458	0.41
26.07	α-Caryophyllene	1458	7.50
27.71	(E,E)-α-Farnesene	1452	0.11
29.45	(+)-δ-Cadinene	1505	0.77
30.11	Caryophyllenyl alcohol	1570	0.16
30.49	(-)-Caryophyllene oxide	1582	2.86
31.48	α-Pinene oxide	932	0.22
32.36	1,3-Bis-(2-cyclopropyl,2-methylcyclopropyl)-but-2-en-1-one	-	0.24
33.02	Isoaromadendrene epoxide	-	0.41
33.43	Patchoulane	-	0.17
53.31	Phenol, 2,2'-methylenebis[6-(1,1-dimethylethyl)-4-methyl-	-	0.30

Table S2. Chemical constituents and their relative abundance by GC-MS in cypress C3 essential oil

RT/min	Compound	RI	Relative abundance (%)
22.49	α-Longipinene	1350	0.81
23.48	Di-epi-α-cedrene	1574	0.30
23.73	Isolongifolene	1389	0.28
23.80	(+)-Sativene	1390	0.53
24.30	(+)-Longifolene	1407	11.80
24.50	(-)-α-Cedrene	1404	0.83
24.72	(-)-Isocaryophyllene	1408	6.00
25.75	α-Caryophyllene	1452	2.13
26.27	Thujopsene	1429	1.11
26.41	Aromadendrene	1458	6.37
27.04	γ-Elemene	1434	9.69
27.38	(+)-Cuparene	1504	9.75
27.62	α-Chamigrene	1503	15.70
27.97	(+)-δ-Cadinene	1522	21.26
29.36	Diepi-α-cedrene epoxide	1402	1.58
30.85	Cedrol	1600	4.46
31.57	Isolongifolanone	1612	4.29
32.17	Torreyol	1644	1.81
32.45	β-Eudesmol	1792	1.28

Table S3. Chemical constituents and their relative abundance by GC-MS in dill D1 weed essential oil

RT/min	Compound	RI	Relative abundance (%)
7.71	(E)-3,7-dimethylocta-1,3,6-triene	1044	0.83
9.96	α-Phellandrene	1002	3.19
10.61	p-Cymene	1020	3.46
10.74	D-Limonene	1024	24.77
16.42	Z-3,3-dimethyl-1-cyclohexaneacetaldehyde	1217	8.37
16.95	(+)-Dihydrocarvone	1191	3.21
17.26	D-(+)-Carvone	1239	56.16

Table S4. Chemical constituents and their relative abundance by GC-MS in geranium G1 essential oil

RT/min	Compound	RI	Relative abundance (%)
12.15	Dihydromyrcenol	1069	0.71
13.05	Linalool	1095	1.74
15.03	DL-Menthone	1148	0.53
16.68	α-Terpineol	1186	0.41
18.26	Citronellol	1223	6.56
19.31	Nerol	1227	2.86
22.77	(+)-α-Longipinene	1350	3.08
23.03	(+)-Calarene	1374	4.43
23.34	(+)-Cycloisosativene	-	1.16
23.45	(+)-Longicyclene	1371	2.88
23.60	Ylangene	1373	1.48
24.02	(-)-Isolongifolene	1389	9.14
24.59	(+)-Longifolene	1407	15.87
24.73	Isosativene	-	8.59
25.00	(-)-Isocaryophyllene	1408	30.50
25.25	α-Selinene	1498	0.54
25.88	Caryophyllene	1417	4.15
26.02	α-Caryophyllene	1452	1.06
27.50	**α-Muurolene**	1500	1.62
27.74	(S)-β-Bisabolene	1505	0.74
28.28	(+)-δ-Cadinene	1522	0.58
30.53	(-)-Caryophyllene oxide	1582	1.35

Table S5. Chemical constituents and their relative abundance by GC-MS in lavender L1 essential oil

RT/min	Compound	RI	Relative abundance (%)
10.76	D-Limonene	1024	0.22
10.83	Eucalyptol	1026	1.16
12.15	Dihydromyrcenol	1069	1.81
13.09	Linalool	1095	22.20
14.24	1,2-Dihydrolinalool	1131	0.20
14.64	L-(-)-Camphor	1141	1.22
15.54	(±)-Borneol / (±)-Isoborneol	1155	0.18
15.86	3,5,5-Trimethychexylacetate	1703	1.72
19.34	3,7-Dimethyl-1,6-octadien-3-yl 2-aminobenzoate	-	18.81
20.30	1-methyl-4-(1-methylvinyl)cyclohexyl acetate	-	0.65
20.50	DL-Isoborneol	1155	0.34
22.69	Terpinyl acetate	1346	4.68
22.85	Triacetin	1347	46.51
24.39	Diphenyl ether	-	0.15
25.53	(-)-Isosativene	1365	0.13

Table S6. Chemical constituents and their relative abundance by GC-MS in niaouli N1 essential oil

RT/min	Compound	RI	Relative abundance (%)
7.53	α-Phellandrene	1002	0.47
7.73	DL-α-Pinene	932	1.89
8.98	(±)-Sabinene	969	2.72
9.08	β-Pinene	974	1.09
10.62	p-Cymene	1020	7.63
10.83	Eucalyptol	1026	1.08
11.74	γ-Terpinene	1054	13.58
12.17	cis-Linalool oxide (furanoid)	1067	0.51
12.69	4-Methyl-3-(1-methylethylidene)-cyclohexene	-	4.68
13.05	Linalool	1095	4.23
13.22	pentylcvalerate	-	0.52
14.45	(-)-trans-pinocarveol	1135	3.13
14.65	L-(-)-Camphor	1141	5.72
16.10	(±)-4-Terpineol	1174	48.46
16.69	α-Terpineol	1186	3.71
25.59	Aromadendrene	1458	0.59

Table S7. Chemical constituents and their relative abundance by GC-MS in origanum O1 essential oil

RT/min	Compound	RI	Relative abundance (%)
6.54	o-Xylene	-	0.12
7.55	α-Phellandrene	1002	0.13
7.58	(+)-Ledene	1496	0.11
7.58	α-Thujene	924	0.16
7.77	α-Cubebene	1345	1.63
8.23	Camphene	946	0.47
9.34	β-Pinene	974	1.01
9.59	β-Myrcene	988	1.26
9.96	3-Carene	1008	0.14
10.00	α-Gurjunene	1409	0.12
10.41	Terpinene	1014	0.57
10.67	p-Cymene	1020	12.63
10.80	Limonene	1024	1.36
10.88	Eucalyptol	1026	0.26
11.79	α-Terpinene	1014	2.17
12.07	β-Terpineol	1159	0.10
12.74	Terpinolene	1086	0.44
12.75	γ-Terpineol	1199	0.40
13.10	Linalool	1095	4.16
15.62	L-(-)-Borneol	1165	0.35
15.66	Borneol	1165	0.37
16.16	Terpinen-4-ol	1174	0.36
16.77	α-Terpineol	1186	0.18
18.96	Patchoulane	1232	0.71
20.82	Thymol	1298	5.03
21.20	Carvacrol	1298	61.10
25.07	γ-Elemene	1434	1.51
27.80	β-Bisabolene	1505	0.41
30.62	Viridiflorol	1592	0.68
33.21	Octanal	998	0.54
36.44	(-)-α-Copaene	1374	0.78
37.75	Isoaromadendrene epoxide	-	0.23
40.25	3-Benzylsulfonyl-2,6,6-trimethylbicyclo(3.1.1)heptane	-	0.19
40.97	Tetradecanoic acid	1795	0.34

Table S8. Chemical constituents and their relative abundance by GC-MS in vanilla V1 essential oil

RT/min	Compound	RI	Relative abundance (%)
10.76	D-Limonene	1024	0.81
14.65	Dihydroterpineol	-	0.42
14.99	Citronellal	1148	13.45
16.69	α-Terpineol	1186	1.96
18.28	Citronellol	1223	2.62
19.33	Geraniol	1249	2.37
22.77	(+)-α-Longipinene	1350	3.51
23.34	(+)-Cycloisosativene	-	0.73
23.45	(+)-Longicyclene	1371	3.10
23.61	α-Copaene	1374	0.54
24.08	(+)-Sativene	1390	2.58
24.59	(+)-Longifolene	1407	43.84
25.00	(-)-Isocaryophyllene	1408	16.64
25.59	(-)-Alloaromadendrene	1458	0.51
26.02	α-Caryophyllene	1452	2.92
26.88	(-)-Germacrene D	1480	1.05
28.30	(+)-δ-Cadinene	1522	1.21
29.25	α-Elemol	1548	1.08
30.54	(-)-Caryophyllene oxide	1582	0.65

Table S9. Chemical constituent and their relative abundance by GC-MS in tea tree T1 essential oil

RT/min	Compound	RI	Relative abundance (%)
7.71	DL-α-Pinene	932	1.18
10.36	α-Terpinene	1014	0.52
10.62	p-Cymene	1020	0.42
10.81	Eucalyptol	1026	1.24
11.73	γ-Terpinene	1054	2.04
14.44	(-)-trans-pinocarveol	1135	0.46
14.63	L-(-)-Camphor	1141	0.86
16.06	(±)-4-Terpineol	1174	28.96
16.66	α-Terpineol	1186	5.34
22.69	Terpinyl acetate	1346	1.76
24.68	(-)-α-Gurjunene	1409	1.94
24.99	(-)-Isocaryophyllene	1408	1.81
25.23	(-)-Alloaromadendrene	1458	0.54
25.38	(+)-Calarene	1431	1.19
25.58	(-)-Aromadendrene	1458	29.90
26.01	α-Caryophyllene	1452	1.61
27.04	(+)-β-Selinene	1498	0.87
27.12	δ-Guaiene	1437	2.01
27.32	(+)-Ledene	1496	3.92
27.47	α-Muurolene	1500	0.49
28.18	Aromadendrene, dehydro-	1460	0.49
28.27	(+)-δ-Cadinene	1522	0.65
29.67	Epiglobulol	-	1.31
29.95	Viridiflorol	1592	0.58
30.55	Globulol	1590	3.66
30.83	Ledol	1602	0.95
32.15	γ-Eudesmol	1630	0.98
32.84	α-Eudesmol	1652	4.30

Table S10. Chemical constituents and their relative abundance by GC-MS in sweet orange S4 essential oil

RT/min	Compound	RI	Relative abundance (%)
7.62	α-Pinene	932	0.41
8.87	β-Pinene	974	0.25
9.44	β-Myrcene	988	1.35
10.04	β-Ocimene	1044	0.10
10.76	D-Limonene	1024	78.25
12.93	Linalool	1095	0.45
14.09	(+)-Limonene oxide	1132	0.31
14.26	(+)-trans-Limonene oxide	1137	0.17
16.53	(S)-(-)-α-Terpineol	1186	0.07
16.75	Perilla alcohol	1294	0.04
17.20	Decanal	1201	0.08
18.77	Carvone	1239	0.10
19.03	Oxalic acid	-	0.03
19.12	2,4-dimethylundecane	-	1.37
20.96	n-Tridecane	1300	6.17
22.57	Dodecane	1200	3.76
23.96	2,6-dimethyloctane	930	0.54
24.51	2,6-dimethylundecane	-	2.28
25.74	2-Methyltridecane	-	4.29
